# Establishment of a model for predicting preterm birth based on the machine learning algorithm

**DOI:** 10.1186/s12884-023-06058-7

**Published:** 2023-11-10

**Authors:** Yao Zhang, Sisi Du, Tingting Hu, Shichao Xu, Hongmei Lu, Chunyan Xu, Jufang Li, Xiaoling Zhu

**Affiliations:** 1 School of Nursing, Wenzhou Medical University, Wenzhou, Zhejiang China; 2 People’s Hospital of Deyang City, Deyang, Sichuan China; 3 The Second Affiliated Hospital of Wenzhou Medical University, Wenzhou, Zhejiang China; 4 Hangzhou Hospital of Traditional Chinese Medicine, Hangzhou, Zhejiang China; 5 Wenzhou Manna Medical Technology Ltd, Wenzhou, Zhejiang China

**Keywords:** Electronic health records, Machine learning, Preterm birth, Prediction, Risk factors of preterm birth

## Abstract

**Background:**

The purpose of this study was to construct a preterm birth prediction model based on electronic health records and to provide a reference for preterm birth prediction in the future.

**Methods:**

This was a cross-sectional design. The risk factors for the outcomes of preterm birth were assessed by multifactor logistic regression analysis. In this study, a logical regression model, decision tree, Naive Bayes, support vector machine, and AdaBoost are used to construct the prediction model. Accuracy, recall, precision, F1 value, and receiver operating characteristic curve, were used to evaluate the prediction performance of the model, and the clinical application of the model was verified.

**Results:**

A total of 5411 participants were included and were used for model construction. AdaBoost model has the best prediction ability among the five models. The accuracy of the model for the prediction of “non-preterm birth” was the highest, reaching 100%, and that of “preterm birth” was 72.73%.

**Conclusions:**

By constructing a preterm birth prediction model based on electronic health records, we believe that machine algorithms have great potential for preterm birth identification. However, more relevant studies are needed before its application in the clinic.

## Introduction

Preterm birth, defined as a live baby born before 37 weeks of completed gestation, is the main factor in 35% of infant mortality [1, 2]. Each year, nearly 15 million infants are born prematurely worldwide, and more than 1 million die from preterm birth and its complications before the age of 5 [3]. Of note, however, even those who survive are at significantly increased risk of disability [4] as well as a range of health problems such as diabetes [5], hypertension [6] and heart disease [7]. Survivors of preterm birth require frequent medical care, which adversely affect their quality of life and mental health, thus imposes an additional burden on the family economy and health care system [8]. Therefore, early identification of preterm birth before its occurrence is crucial to help health care providers offer timely interventions to improve neonatal outcomes.

No standardized criteria have been developed regarding the prediction of preterm birth due to the complexity of the mechanism [9]. Currently, clinical assessment of preterm birth is mainly based on risk factor assessment, cervical measurement, and biochemical marker assessment [10, 11]. However, despite their potential to identify specific preterm birth characteristics, not all of these assessment measures are therapeutically applicable to every pregnant woman due to a lack of safety or cost-effectiveness [12]. For example, tests such as biochemical marker assessment and cervical measurement will place additional physical and psychological stress on the pregnant woman, meanwhile the cost of the tests increases the financial strain on the family [13].

An ongoing trend is to carry out an initial assessment of patient prognosis based on electronic health records (EHRs). EHRs are large, readily available, real-time databases that can be used to predict diseases [14], and their feasibility has been proven in a variety of diseases such as hypertension [15], heart disease [16], sepsis [17] and mental disorders [18]. In the field of preterm birth, the potential for the use of EHRs is even more striking. According to the standards of obstetric care, many countries advocate strongly for women to receive regular maternal examinations during pregnancy to ensure the safety. This enables more regular, more standardized, and more comprehensive recording of information on pregnant women in EHRs compared to other patients [19]. Within the factors related with preterm birth, a substantial proportion are documented in the EHRs, including (1) maternal characteristics: parturient woman age, abortion, hypertension, diabetes, anemia, uterine abnormalities, scar uterus, pregnancy complicated with uterine fibroids, colonization of streptococcus agalactiae [20]; (2) fetal factors: abnormal weight, number of neck loops, fetal distress [21]; (3) current pregnancy: parity, placenta previa, preterm premature rupture of membranes, multiple pregnancies, abnormal fetal position, prenatal hemorrhage, abnormal amniotic fluid volume, assisted pregnancy [22]. However, it is impractical to manually analyze large amounts of data across many pregnant women to make the correct decisions and advance interventions in a busy clinical setting. Manual assessment requires healthcare workers with high professional level, and due to the influence of the subjective judgment of healthcare workers, there is a higher risk of error.

Machine learning, a branch of artificial intelligence, can prospectively predict patient clinical outcomes by extracting information from data, and has been increasingly used in the clinical settings in recent years [23]. Currently, there are also some studies using machine learning in the prediction of preterm birth [24]. However, in most studies, the datasets are relatively small and the input data used to predict preterm birth varies [25]. For example, H M, B M, S O, V F, K M, B C and J B [26] used the metabolic panels in amniotic fluid, while L Chen and Y Hao [27] used the images of electro-hysterogram (EHG). The exploration of models that can be transformed and applied to clinical practice is still limited. [28].

Therefore, in this research, relevant information was extracted from the EHRs for the construction of a preterm birth prediction model. By constructing the model and interpreting its internals, a reference is provided for the development of a preterm birth prediction model that can be put into clinical practice.

## Methods

### Study design and dataset

This study used a retrospective cohort study design. The dataset comprised information on deliveries at the Second Hospital of Wenzhou Medical University from October 2019 to December 2020. To ensure data availability, mothers without complete medical records were excluded. Because of the lack of a specific sample size, which could lead to bias in the results, women with systemic lupus erythematosus and psychiatric disorders and those who delivered malformed fetuses or stillborn fetus were excluded, although these may also be influential factors in preterm birth to some extent. This study was approved by the Ethics Committee of Wenzhou Medical University (No. 2,019,099). As a retrospective study, individual data were processed in this article in an anonymous way, and each piece of data remains unlinked to any individual, therefore consent was waived for this study with the permission of Wenzhou Medical University.

### Data collection and preprocessing

Through literature review and expert group meetings, 21 factors that may be risk factors of preterm birth were preliminaries identified, among which parity and number of neck loops were numerical variables, parturient woman age, abortion, placenta previa, preterm premature rupture of membranes, hypertension, diabetes, anemia, multiple pregnancies, abnormal fetal position, prenatal hemorrhage, uterine abnormalities, scar uterus, pregnancy complicated with uterine fibroids, fetal distress, colonization of streptococcus agalactiae, amniotic fluid traits, abnormal amniotic fluid volume, assisted pregnancy and fetal weight abnormality were classified variables. The above data were extracted from the electronic health record system, and the modeling variables were screened by unconditional multi-factor logistic regression.

In this study, the dataset contained 119,042 records, and the number of deletion cases was 12 (including 5 cases of Parity, 6 cases of number of neck loops and 1 case of hypertension). Parity and number of neck loops are continuous numerical data, which can be used for curve fitting. The values on the curve were selected by random number table method to fill the missing values. As the number of missing cases of hypertension is too small and the interpolation fitting effect is not good, it was deleted.

### Outcomes

The dependent variable was the outcome of preterm birth and was dichotomized into binary outcomes as 0 and 1. Thus, preterm birth was recorded as 1, and non-preterm birth was recorded as 0.

### Screening of modeling variables

In this study, the non-conditional logistic regression method was used to select statistically significant independent variables by univariate and multivariate analysis; the outcome of labor was the dependent variable, and 21 suspected factors of preterm birth were independent variables. The variables with statistical significance (P < 0.10) were analyzed by non-conditional multivariate analysis, and those with statistical significance (P < 0.10) in the non-conditional multivariate analysis were included as modeling variables (Table 1).

**Table 1:** Maternal Characteristics and the analysis results of logistic regression

Variables	Non-preterm birth(N = 4778)	Preterm birth(N = 633)	Univariate analysis		Multivariate analysis	
			P	OR (95%CI)	P	OR (95%CI)
**Parity**	1.37 ± 0.68	1.49 ± 0.80	0.23	0.88(0.71~1.09)		
**Number of neck loops**	0.38 ± 0.57	0.29 ± 0.53	0.15	0.83(0.64~1.07)		
**Parturient woman age**			0.02	1.59(1.07~2.38)	0.05	1.48(1.00~2.19)
< 20 or > 35	453(79.3%)	118(20.7%)				
≥ 20 and ≤ 35	4325(89.4%)	515(10.6%)				
**Abortion**			0.63	0.87(0.50~1.52)		
≥ 3 or adverse pregnancy history was 1	245(82.5%)	52(17.5%)				
<3	4533(88.6%)	581(11.4%)				
**Placenta previa**			<0.01	4.62(1.86~11.52)	<0.01	4.24(1.72~10.44)
Yes	21(30%)	49(70%)				
No	4757(89.1%)	584(10.9%)				
**Preterm premature rupture of membranes**			<0.01	172.36(103.14~288.06)	<0.01	164.76(99.06~274.03)
Yes	21(6.7%)	291(93.3%)				
No	4757(93.3%)	342(6.7%)				
**Hypertension**			0.99	0.99(0.48~2.08)		
Yes	108(73.5%)	39(26.5%)				
No	4670(88.7%)	594(11.3%)				
**Diabetes**			0.05	1.46(1.00~2.14)	0.08	1.41(0.96~2.05)
Yes	448(75.3%)	147(24.7%)				
No	4330(89.9%)	486(10.1%)				
**Multiple pregnancies**			<0.01	40.76(21.57~77.04)	<0.01	41.90(22.91~76.63)
Yes	21(12.4%)	148(87.6%)				
No	4757(90.7%)	485(9.3%)				
**Abnormal fetal position**			<0.01	2.58(1.72~3.85)	<0.01	2.63(1.76~3.91)
Yes	247(60.2%)	163(39.8%)				
No	4531(90.6%)	470(9.4%)				
**Prenatal hemorrhage**			<0.01	6.52(3.59~11.84)	<0.01	6.67(3.68~12.09)
Yes	47(26.3%)	132(73.7%)				
No	4731(90.4%)	501(9.6%)				
**Uterine abnormalities**			0.38	1.95(0.44~8.69)		
Yes	8(61.5%)	5(38.5%)				
No	4770(88.4%)	628(11.6%)				
**Scar uterus**			<0.01	3.82(2.63~5.54)	<0.01	3.55(2.52~5.00)
Yes	391(71.7%)	154(28.3%)				
No	4387(90.2%)	479(9.8%)				
**Pregnancy complicated with uterine fibroids**			0.30	0.73(0.41~1.31)		
Yes	280(81.6%)	63(18.4%)				
No	4498(88.8%)	570(11.2%)				
**Fetal distress**			0.89	1.03(0.72~1.46)		
Yes	273(90.1%)	30(9.9%)				
No	4372(88.3%)	582(11.7%)				
Doubt	133(86.4%)	21(13.6%)				
**Colonization of Streptococcus agalactiae**			0.04	0.48(0.25~0.95)	0.04	0.49(0.25~0.95)
Yes	380(94.5%)	22(5.5%)				
No	4398(87.8%)	611(12.2%)				
**Amniotic fluid traits**			0.01	0.55(0.36~0.85)	0.01	0.56(0.37~0.87)
Abnormality	968(94.8%)	53(5.2%)				
No abnormality	3810(86.8%)	580(13.2%)				
**Abnormal amniotic fluid volume**			0.35	1.27(0.77~2.10)		
No	4607(88.5%)	596(11.5%)				
Too little	160(83.3%)	32(16.7%)				
Too much	11(68.8%)	5(31.3%)				
**Anemia**			0.40	1.19(0.80~1.76)		
Yes	479(80.1%)	119(19.9%)				
No	4299(89.3%)	514(10.7%)				
**Assisted pregnancy**			0.68	0.89(0.52~1.54)		
Yes	253(71.7%)	100(28.3%)				
No	4525(89.5%)	533(10.5%)				
**Fetal weight abnormality**			<0.01	4.89(4.01~5.96)	<0.01	4.92(4.04~5.98)
Normal	4529(93.5%)	317(6.5%)				
Macrosomia	191(90.5%)	20(9.5%)				
Low body weight baby	58(16.4%)	296(83.6%)				

### Establishing premature birth prediction model based on machine learning algorithm

In this study, a total of 5411 cases were divided into a training set comprising 4329 cases and a test set consisting of 1082 cases in an 8:2 ratio. The training set was utilized for model training, while the test set served as internal validation. Experimental results were further validated using 50% fold cross-validation. Prediction models were constructed employing logistic regression, decision tree, Naive Bayes, support vector machine, and AdaBoost algorithms with parameters specified in Table 2.

**Table 2:** The Five model parameter settings

Model	Parameter setting
Logistic Regression Model	Parameters were set to default values
Decision Tree	The node splitting criterion was Gini diversity index, node partition mode was best, the maximum number of splits was 100, the maximum depth of the tree was unlimited, and other parameters were set to default values
Naïve Baye	The function was Gaussian radial kernel function (RBF), the smoothing (alpha value) was 1, and other parameters were set to default values
Support Vector Machine	The penalty coefficient of error term was 1, the kernel was RBF, the kernel coefficient value was 0.01, the multiclassification decision function was over, the model convergence parameter was 0.001, the maximum number of iterations was 2000, and other parameters were set to default values
AdaBoost	The learner type was the decision tree, the maximum number of splits was 20, the number of learners was 30, and the learning rate was 0.1

### Evaluation of the prediction model

The area under a curve (AUC) of a receiver operating characteristic (ROC) curve, sensitivity, specificity, and F1 score were used to evaluate the performance of the model.

### Clinical prospective verification of the prediction model

After comparing the five models, the prediction model with the best performance was chosen for external verification. The data from pregnant women who gave birth in the same hospital from November to and December 2020 were collected. The difference between the actual clinical outcome of premature birth and the decision-making result of the predictive model was comparable, expressed as the accuracy (%).

### Statistical method

SPSS 22.0 was used for data analysis. The 21 influencing factors and the outcome of premature birth were analyzed by logistic single factor analysis. According to the single factor analysis results, a stepwise regression method was utilized to screen the variables; P < 0.1 was regarded as statistically significant. Establish and verify different types of machine learning algorithms built on MATLAB.

## Result

### Baseline characteristics

**Fig. 1:**
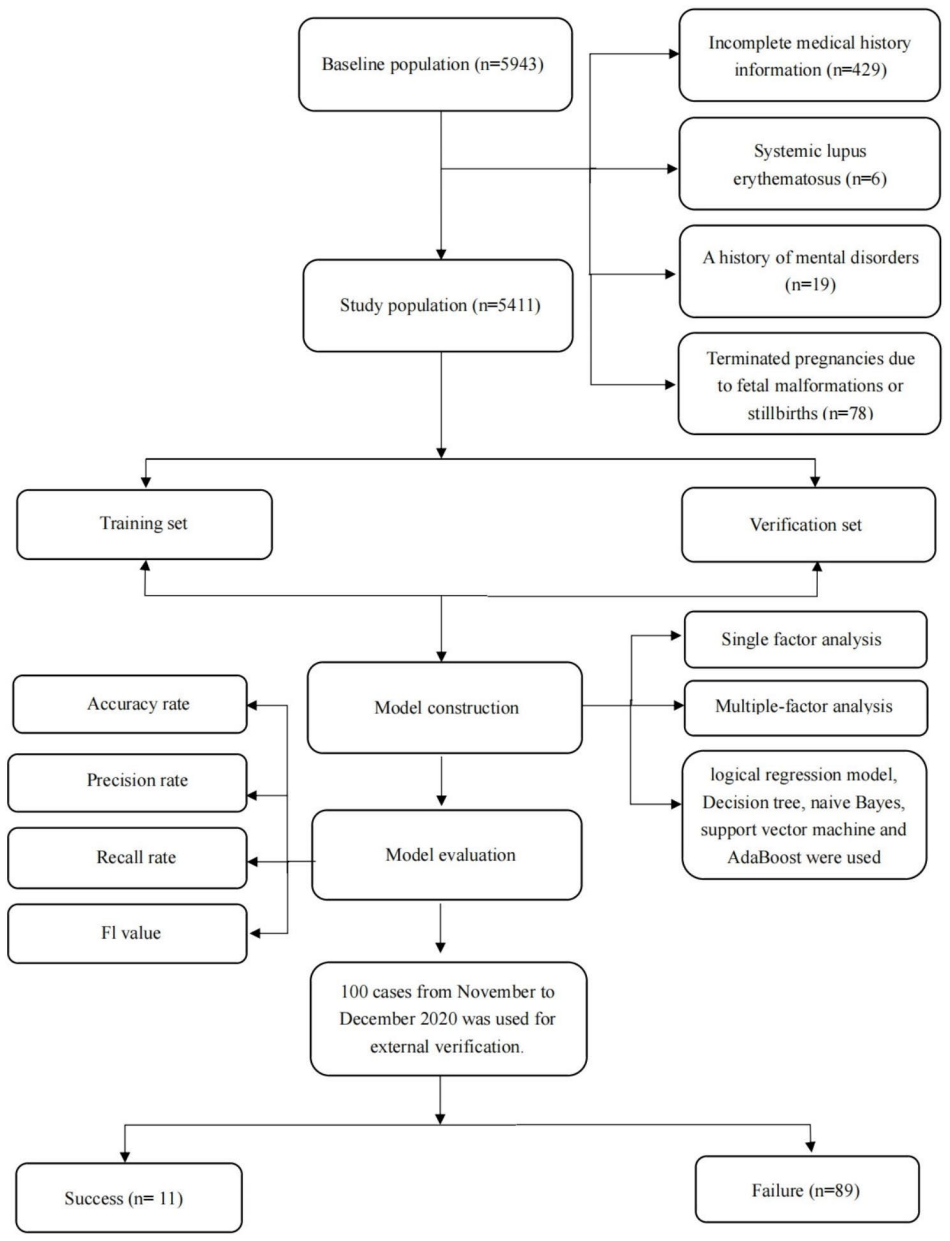
A flow chart for study population selection

Totally, the dataset contained 5943 participants between October 2019 and October 2020. After excluding 429 participants with incomplete medical history information, 6 participants with systemic lupus erythematosus, 19 participants with a history of mental disorders, and 78 participants with terminated pregnancies due to fetal malformations or stillbirths, a total of 5411 participants (633 preterm births and 4778 non-preterm births) were included and used for model construction (Fig. 1).

### Identification of feature importance

Univariate analysis showed that diabetes, parturient woman age, amniotic fluid traits, fetal weight abnormality, abnormal fetal position, scar uterus, colonization of streptococcus agalactiae, prenatal hemorrhage, placenta previa, multiple pregnancies, preterm premature rupture of membranes and preterm birth were significantly related with preterm birth (P < 0.10). The independent variables with statistical significance in univariate analysis were included as predicting factors in the multi-factor unconditional logistic stepwise regression analysis, and finally eleven variables were statistically significant (P < 0.10), (Table 1).

### Prediction results of five models

**Fig. 2:**
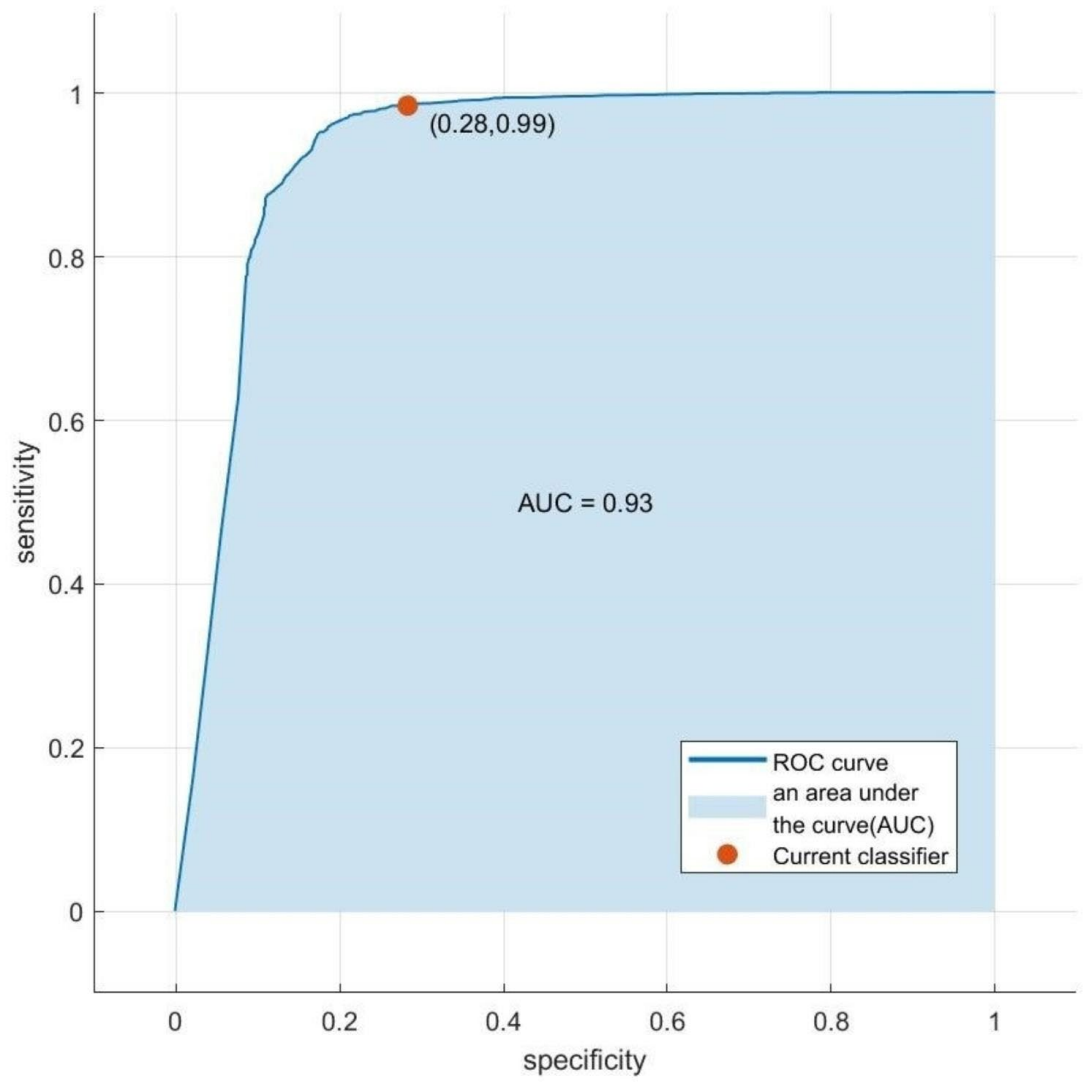
ROC curves of the AdaBoost model

AdaBoost model has the best prediction ability among the five models, with an accuracy of 0.954, a recall rate of 0.985, a precision rate of 0.963, and a F1 value of 0.969, which is obviously better than the other four methods, as shown in Table 3. The AUC of the AdaBoost model was 0.93, as shown in Fig. 2.

**Table 3:** Accuracy, precision, recall rate, and F1 value of the five models

Model	Accuracy	Recall	Precision	F1 value
Logistic Regression Model	0.953	0.987	0.960	0.970
Decision Tree	0.953	0.984	0.964	0.968
Naïve Bayes	0.945	0.964	0.974	0.954
Support Vector Machine	0.949	0.978	0.964	0.963
AdaBoost	0.954	0.985	0.963	0.969

### External verification of the model

The prediction model was clinically verified by data from 100 randomly selected women who gave birth between November and December 2020. Among them, 89 cases were non-preterm birth and 11 cases were preterm birth. The accuracy of the model for the prediction of “non-preterm birth” was the highest, reaching 100%, and that of “preterm birth” was 72.73%. The results of the confusion matrix are shown in Table 4.

**Table 4:** External verification results of prediction model

Actual outcome	AdaBoost		
	Non-preterm birth	Preterm birth	Accuracy rate
**Non-preterm birth**	89	0	100%
**Preterm birth**	3	8	72.73%

### Influence of variables on predictions

**Fig. 3:**
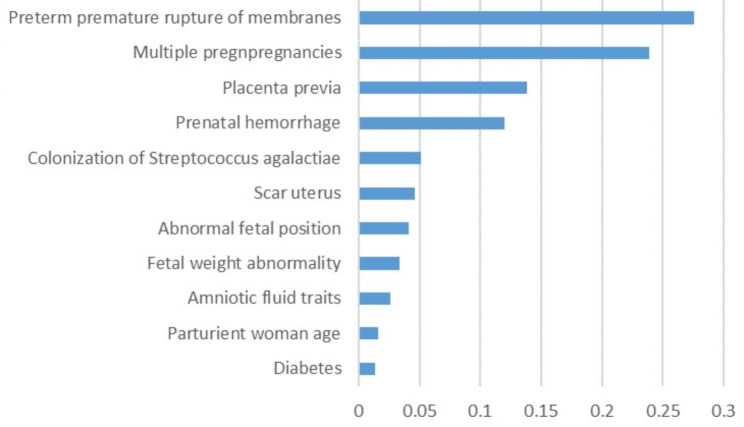
Ranking of variable importance

The distribution of the variables incorporated in the AdaBoost prediction model is shown in Fig. 3. Preterm premature rupture of membranes is the most important risk factor of preterm birth, followed by multiple pregnancies, placenta previa, prenatal hemorrhage, colonization of streptococcus agalactiae, scar uterus, abnormal fetal position, fetal weight abnormality, amniotic fluid traits, parturient woman age, diabetes.

## Discussion

By extracting relevant information from the EHRs, a model that can be used for the initial assessment of preterm labor was identified using machine learning techniques. This study demonstrates the potential of machine learning techniques for predicting preterm birth in busy clinical settings. Furthermore, by analyzing the weights of the influencing factors within the best model, a reference can be provided for the prediction of preterm birth.

In this study, 2 numerical variables and 19 categorical variables were used as independent variables to study the relationship between the binary categorical variable preterm birth, so the possibility of preterm birth was predicted by a binary logistic regression model. To compare with the logistic regression model, decision tree models and support vector machines were utilized due to their ability to directly process non-numerical data and handle missing values. Naive Bayes, known for its stable classification efficiency, was also included in the research. However, the limitations of the above models in the research process, such as decision tree and naive Bayes are not strong in explaining models with large samples were observed, which aligns with the findings of this study. Considering that support vector machines are sensitive to missing data, an AdaBoost model was incorporated into the research. AdaBoost is a high-precision classifier that can construct sub-classifiers using various methods while maintaining simplicity without feature screening or concerns about overfitting. In this study, the prediction accuracy of the five models was relatively high, falling between 0.945 and 0.954 (mean 0.951 ± 0.004), which is higher than the previous average of 0.84 for preterm birth prediction models [25]. Among them, with the highest accuracy of 0.954, AdaBoost has an AUC of 0.93, suggesting that the AdaBoost model was effective in predicting preterm birth and the model fit was good.

Investigation of factors influencing preterm birth is an essential foundation for predicting preterm birth. There are inextricable links between the influencing factors, which makes it harder to elucidate the specific mechanisms by which preterm birth occurs in individuals. In this study, based on variable importance from the AdaBoost, major determinants of preterm birth are preterm premature rupture of membranes, multiple pregnancies, placenta previa, prenatal hemorrhage, colonization of streptococcus agalactiae, scar uterus, abnormal fetal position, fetal weight abnormality, amniotic fluid traits, parturient woman age and diabetes.

Preterm premature rupture of membranes had the highest weight of influencing preterm birth prediction. According to research, only about 8% of women with preterm premature rupture of membranes are able to heal [29]. The ruptured membranes allow the entry of external microorganisms into the amniotic cavity and result in the loss of amniotic fluid, which reduces the buffering capacity of the uterine wall to protect the fetus [30]. As a result, it leads to an increased risk of various complications such as infection and fetal distress, which in turn further endangers pregnancy outcomes [31, 32].

Consistent with previous studies, multiple pregnancies appear to be another important influencing factor for predicting preterm birth [33]. It has been shown that multiple pregnancies overstretch the uterus due to the larger space required for multiple fetuses, which can lead to uterine contractions [34]. This may raise the incidence of complications such as intrauterine growth restriction, abnormal fetal position, and intrauterine distress [35, 36]. In addition, for pregnant women, multiple births exacerbate the pressure on the circulation, thus increasing the risk of hypertension during pregnancy [37]. All of these changes may increase the risk of preterm birth.

Placenta praevia and prenatal hemorrhage are also relatively important factors in predicting preterm labor. Prenatal hemorrhage can be caused by a variety of factors, such as injury, excessive oxytocin, obstructed labor, scarred uterus, multiple pregnancies, excessive amniotic fluid, fetal malposition, etc. However, the most common causes of prenatal hemorrhage in late pregnancy are placental abruption and placenta praevia [38]. In women with anterior placenta, when the lower part of the uterus is extended, the placenta has difficulty in changing accordingly, causing displacement between them, which reduces the effective area of the placenta and thus limits the oxygen exchange and nutrient availability to the fetus. In addition, placenta praevia can easily be combined with placental implantation, which also increases the risk of hemorrhage and greatly affects the blood circulation of the fetus, limiting fetal growth and leading to preterm labor [39].

Based on the results of AdaBoost in this study, it is imperative to remain vigilant regarding preterm premature rupture of membranes, placenta praevia, and prenatal hemorrhage. Pregnant women should be educated about identifying relevant risk symptoms to ensure timely medical assistance when early symptoms arise, thereby minimizing the likelihood of adverse pregnancy outcomes. Furthermore, greater attention needs to be devoted to managing the health of women with multiple pregnancies. They should adhere strictly to healthy diets and engage in moderate exercise in order to effectively control weight so as to prevent uterine overextension, which could potentially increase the risk of adverse pregnancy outcomes.

Previously, researchers have developed machine learning-based models for predicting preterm birth. For instance, T Khatibi, N Kheyrikoochaksarayee and MM Sepehri [40] constructed a preterm birth prediction model by incorporating 112 variables such as demographic information (e.g., age, residence, education level), disease history (hypertension, diabetes, miscarriage), and pregnancy conditions (placenta previa, premature rupture of membranes). However, the AUC of their final model was 0.68 which is lower than this study. This suggests that the number of variables used in model construction may not be critical; instead, it is more important to select influential factors highly correlated with preterm birth. Rawashdeh H [41] utilized 19 input variables to develop a predictive model for preterm birth using algorithms such as decision trees and random forests, achieving an AUC of 0.97, slightly higher than the present study. However, their inclusion of cervical length as a variable increased the burden on pregnant women during examinations. In contrast, this study constructed a safer and more convenient model by utilizing variables already recorded in EHRs at the time of obstetric examinations, eliminating the need for additional tests. Furthermore, few previous studies have conducted external validation when developing machine learning prediction models for preterm birth. In this study, on the basis of internal validation of the optimal model, external validation of the model was performed with a test dataset to further verify the feasibility.

The practical implementation of this model requires the integration of various resources and still faces challenges. Data based solely on EHRs inevitably mask characteristics that are prone to temporal fluctuations, such as blood glucose and blood pressure. This may explain the insignificant effect of blood pressure on preterm labor and the relatively low ranking of the importance of blood glucose in this study. Therefore, in practical clinical applications, healthcare professionals can leverage the convenience of the Internet or telephone to regularly collect and update dynamic maternal information, thereby enriching the data source of predictive models. Furthermore, establishing an e-platform based on the preterm labor prediction model would facilitate seamless information exchange and data collection. Pregnant women could timely upload and update relevant indicators while healthcare professionals integrate these new inputs with medical records to enhance preterm labor prediction using this model. However, it is important to acknowledge that despite its utility, this current model may still exhibit inaccuracies in predicting a small subset of cases; hence further refinement through collaboration with experienced physicians is warranted.

One limitation of this study is the potential for retrospective leakage, as it relies heavily on EHRs. The reliability of EHRs is constrained by the frequency of maternal contact with the hospital, such as prenatal check-ups and hospitalizations, which may introduce irregularity in data sources. Additionally, variations in recording methods and data accuracy due to different recording personnel involved could lead to biased results and undermine the representativeness of the data in reflecting true clinical situations. Our current model lacks specification regarding its predictive accuracy for individual preterm births at different stages of pregnancy since it combines comprehensive data across an entire period of pregnancy. It is challenging to ensure that a model constructed based on such extensive data would exhibit consistent predictive accuracy in practical applications. Furthermore, using external validation derived from similar EHRs limits the generalizability and discriminatory validity when applying our model to other EHR systems. Given these limitations inherent in retrospective studies, future research should focus on prospective studies to further explore the application of machine learning techniques in predicting preterm birth.

In this study, relevant information was extracted from the EHRs and used to construct a machine learning model for preterm birth prediction. All 21 parameters entered in our model can be obtained from EHRs. The model further confirms that preterm premature rupture of membranes, multiple pregnancies, placenta previa, prenatal hemorrhage, and colonization of streptococcus agalactiae are high-risk factors for preterm birth. However, possibly due to an imbalance in the number of preterm and non-preterm births in the dataset, 30% of actual preterm births were incorrectly assessed as non-preterm births by our model during external validation. We believe that these types of errors can be addressed by collecting data from multiple centers with larger data sizes. In a word, the feasibility of using machine learning techniques to predict preterm birth has been explored, yet before its real application in clinical settings, more expertise is needed and more scholars need to exploit a more systematic application model.

## Data Availability

Supplementary material related to this article can be found, in the online version, at du, sisi (2023), “Establishment of a model for predicting preterm birth based on the machine learning algorithm”, Mendeley Data, V1, doi: 10.17632/jpbtrskhvg.1.

## Abbreviations

EHRs: Electronic health records
EHG: Electro-hysterogram
AUC: Area under a curve
ROC: Receiver operating characteristic

## References

[1] Harrison MS, Goldenberg RL (2016). Global burden of prematurity. Semin Fetal Neonatal Med.

[2] Lancet (2016). The unfinished agenda of preterm births. Lancet.

[3] Walani SR (2020). Global burden of preterm birth. Int J Gynaecol Obstet.

[4] Ion R, Bernal AL (2015). Smoking and Preterm Birth. Reprod Sci.

[5] Crump C, Sundquist J, Sundquist K (2020). Preterm birth and risk of type 1 and type 2 Diabetes: a national cohort study. Diabetologia.

[6] Crump C, Sundquist J, Sundquist K (2020). Risk of Hypertension into adulthood in persons born prematurely: a national cohort study. Eur Heart J.

[7] Greer C, Troughton RW, Adamson PD, Harris SL (2022). Preterm birth and cardiac function in adulthood. Heart.

[8] Baumann N, Bartmann P, Wolke D. Health-Related Quality of Life Into Adulthood after very Preterm Birth. Pediatrics 2016, 137(4). 10.1542/peds.2015-3148 27016272

[9] Medicine FWGoGCPiM-F (2019). Good clinical practice advice: prediction of preterm labor and preterm premature rupture of membranes. Int J Gynaecol Obstet.

[10] Iams JD, Goldenberg RL, Meis PJ, Mercer BM, Moawad A, Das A, Thom E, McNellis D, Copper RL, Johnson F (1996). The length of the cervix and the risk of spontaneous premature delivery. National Institute of Child Health and Human Development Maternal Fetal Medicine Unit Network. N Engl J Med.

[11] Sharifi-Heris Z, Laitala J, Airola A, Rahmani AM, Bender M (2022). Machine Learning Approach for Preterm Birth Prediction Using Health Records: systematic review. JMIR Med Inform.

[12] Oskovi Kaplan ZA, Ozgu-Erdinc AS (2018). Prediction of Preterm Birth: maternal characteristics, ultrasound markers, and biomarkers: an updated overview. J Pregnancy.

[13] Crespi S, Hu XH, Johnson B, Griesinger G, Ledger WL, Heisel O, Kolibianakis EM (2022). Patient burden and healthcare resource utilization of regimens for ovarian stimulation. Reprod Biomed Online.

[14] Abul-Husn NS, Kenny EE (2019). Personalized medicine and the Power of Electronic Health Records. Cell.

[15] Ye C, Fu T, Hao S, Zhang Y, Wang O, Jin B, Xia M, Liu M, Zhou X, Wu Q (2018). Prediction of Incident Hypertension within the Next Year: prospective study using Statewide Electronic Health Records and Machine Learning. J Med Internet Res.

[16] Hyland SL, Faltys M, Hüser M, Lyu X, Gumbsch T, Esteban C, Bock C, Horn M, Moor M, Rieck B (2020). Early prediction of circulatory failure in the intensive care unit using machine learning. Nat Med.

[17] Yan MY, Gustad LT, Nytrø Ø (2022). Sepsis prediction, early detection, and identification using clinical text for machine learning: a systematic review. J Am Med Inform Assoc.

[18] Garriga R, Mas J, Abraha S, Nolan J, Harrison O, Tadros G, Matic A (2022). Machine learning model to predict mental health crises from electronic health records. Nat Med.

[19] Paquette AG, Hood L, Price ND, Sadovsky Y. Deep phenotyping during pregnancy for predictive and preventive medicine. Sci Transl Med 2020, 12(527). 10.1126/scitranslmed.aay1059 PMC7268934 31969484

[20] Muglia LJ, Benhalima K, Tong S, Ozanne S (2022). Maternal factors during pregnancy influencing maternal, fetal, and childhood outcomes. BMC Med.

[21] Goldenberg RL, Culhane JF, Iams JD, Romero R (2008). Epidemiology and causes of preterm birth. Lancet.

[22] Koullali B, Oudijk MA, Nijman TA, Mol BW, Pajkrt E (2016). Risk assessment and management to prevent preterm birth. Semin Fetal Neonatal Med.

[23] Obermeyer Z, Emanuel EJ (2016). Predicting the Future - Big Data, Machine Learning, and Clinical Medicine. N Engl J Med.

[24] Iftikhar P, Kuijpers MV, Khayyat A, Iftikhar A, DeGouvia De Sa M (2020). Artificial Intelligence: a New Paradigm in Obstetrics and Gynecology Research and Clinical Practice. Cureus.

[25] Akazawa M, Hashimoto K (2022). Prediction of preterm birth using artificial intelligence: a systematic review. J Obstet Gynaecol.

[26] B MHMBOSFVMKCB (2022). Metabolomic profiles of mid-trimester amniotic fluid are not associated with subsequent spontaneous preterm delivery or gestational duration at delivery. J maternal-fetal Neonatal Medicine: Official J Eur Association Perinat Med Federation Asia Ocean Perinat Soc Int Soc Perinat Obstetricians.

[27] Chen L, Hao Y (2017). Feature extraction and classification of EHG between Pregnancy and Labour Group Using Hilbert-Huang Transform and Extreme Learning Machine. Comput Math Methods Med.

[28] Yang Q, Fan X, Cao X, Hao W, Lu J, Wei J, Tian J, Yin M, Ge L (2023). Reporting and risk of bias of prediction models based on machine learning methods in preterm birth: a systematic review. Acta Obstet Gynecol Scand.

[29] Lee SM, Park KH, Hong S, Kim YM, Park YH, Lee YE, Jeon SJ (2020). Identification of cultivable Bacteria in amniotic fluid using Cervicovaginal fluid protein microarray in Preterm premature rupture of membranes. Reprod Sci.

[30] Park JW, Park KH, Lee JE, Kim YM, Lee SJ, Cheon DH (2019). Antibody Microarray Analysis of Plasma Proteins for the prediction of histologic chorioamnionitis in women with preterm premature rupture of membranes. Reprod Sci.

[31] Packard RE, Mackeen AD (2015). Labor induction in the patient with preterm premature rupture of membranes. Semin Perinatol.

[32] Skupski D (2019). Preterm premature rupture of membranes (PPROM). J Perinat Med.

[33] Zhang YJ, Shen J, Lin SB, Lu C, Jiang H, Sun Y, Cheng X, Wang H, Cui S, Liu X (2022). The risk factors of preterm birth: a multicentre case-control survey in China in 2018. J Paediatr Child Health.

[34] Norwitz ER, Edusa V, Park JS (2005). Maternal physiology and Complications of multiple pregnancy. Semin Perinatol.

[35] Buca D, Pagani G, Rizzo G, Familiari A, Flacco ME, Manzoli L, Liberati M, Fanfani F, Scambia G, D’Antonio F (2017). Outcome of monochorionic twin pregnancy with selective intrauterine growth restriction according to umbilical artery doppler flow pattern of smaller twin: systematic review and meta-analysis. Ultrasound Obstet Gynecol.

[36] D’Alton M, Breslin N (2020). Management of multiple gestations. Int J Gynaecol Obstet.

[37] Narang K, Szymanski LM (2020). Multiple gestations and Hypertensive disorders of pregnancy: what do we know?. Curr Hypertens Rep.

[38] Silver RM (2015). Abnormal placentation: Placenta Previa, Vasa Previa, and Placenta Accreta. Obstet Gynecol.

[39] Ananth CV, Berkowitz GS, Savitz DA, Lapinski RH (1999). Placental abruption and adverse perinatal outcomes. JAMA.

[40] Khatibi T, Kheyrikoochaksarayee N, Sepehri MM (2019). Analysis of big data for prediction of provider-initiated preterm birth and spontaneous premature deliveries and ranking the predictive features. Arch Gynecol Obstet.

[41] Rawashdeh H, Awawdeh S, Shannag F, Henawi E, Faris H, Obeid N, Hyett J (2020). Intelligent system based on data mining techniques for prediction of preterm birth for women with cervical cerclage. Comput Biol Chem.

